# It is time to talk about people: a human-centered healthcare system

**DOI:** 10.1186/1478-4505-8-35

**Published:** 2010-11-26

**Authors:** Meghan M Searl, Lea Borgi, Zeina Chemali

**Affiliations:** 1 Brigham & Women's Hospital, 221 Longwood Ave., Boston, MA 02115, USA; 2 Harvard Medical School, 25 Shattuck St., Boston, MA 02115, USA; 3 St. Elizabeth's Hospital, 736 Cambridge St., Boston, MA 02135, USA; 4 Massachusetts General Hospital, 55 Fruit St., Boston, MA 02114, USA

## Abstract

Examining vulnerabilities within our current healthcare system we propose borrowing two tools from the fields of engineering and design: a) Reason's system approach [1] and b) User-centered design [2,3]. Both approaches are human-centered in that they consider common patterns of human behavior when analyzing systems to identify problems and generate solutions. This paper examines these two human-centered approaches in the context of healthcare. We argue that maintaining a human-centered orientation in clinical care, research, training, and governance is critical to the evolution of an effective and sustainable healthcare system.

## Introduction

With healthcare reform in the spotlight there exists a window of opportunity to identify weaknesses in our healthcare system and propose workable solutions. At the present time, few would argue that the U.S. Healthcare system functions optimally. There are many reasons for this, including the fragmenting effect of competing interests in the health marketplace with insufficient incentives for building and maintaining systems that knit these fragments together. While the full list of reasons goes beyond the scope of this paper, one critical weakness to understand is that the U.S. Healthcare system at the present time is not inherently human-centered. That is, on many levels, the current healthcare system is not designed for optimal use by human beings. It is when human tendencies are ignored that opportunities for error become insidious.

We propose that a robust and sustainable healthcare system must be human-centered. Efforts to build and rebuild parts of the system in a human-centered fashion require reliable tools. Two of these tools that are currently underutilized in the area of healthcare are a) Reason's system approach [1] and b) User-centered design [2,3]. As a call for more human-centered approaches to optimizing healthcare delivery, we review these two approaches, using a variety of examples to illustrate both real and potential applications.

### Two human-centered tools

In his studies of safety, James Reason made a critical distinction between two ways of understanding why human errors occur: the person approach and the system approach [1]. While the person approach blames the individual most closely linked to the error, the system approach takes into account all of the many contributing factors that played a role in the manifestation of the error.

As a part of the system approach, Reason proposed the "Swiss cheese" model to explain why system failures occur [4]. In this model he suggests that in complex systems, failures are prevented from occurring much of the time because of a variety of barriers that serve as checks and balances. In most systems, however, these barriers have areas of weakness. When a series of weaknesses align, a hole in the system appears such that barriers no longer exist to prevent failure and a breakdown occurs. Latent errors refer to weaknesses in the system that, when combined with relevant stressors, contribute to active failures. Active failures are the unsafe acts committed by people who are in direct contact with the patient or system [4].

According to Reason, "The basic premise in the system approach is that humans are fallible and errors are to be expected, even in the best organizations." What is so striking about this model is that it provides a framework for viewing people as vulnerable, rather than inherently faulty. Reason explicitly acknowledges the existence of identifiable vulnerabilities within and outside of each person that give rise to errors when those vulnerabilities are aligned. So, in this constructive, human-centered approach, he neutralizes any implicit blame by saying that "even the best organizations" are fallible.

While Reason's system approach is a top-down method for analyzing problems, user-centered design is a bottom-up method for developing solutions. The term 'user-centered design' was coined by Donald Norman and became widely adopted after he published User-Centered System Design: New Perspectives on Human-Computer Interaction in 1986 and then The Psychology Of Everyday Things in 1988 [2,3]. User-centered design, of which human-centered design is a specific instance, is a technique used in the field of engineering that prioritizes the relevant characteristics of a product user throughout the design of a product.

Taking the example of designing an automobile dashboard, a user-centered designer would spend a great deal of time in the proverbial shoes of the future driver, trying to understand his needs (*What pieces of information does he need to monitor while driving?*), preferences (*Would he like to have the current radio station displayed at eye level?*) and limitations (*How many pieces of information can he process simultaneously?*), among other things. Like Reason's system approach, user-centered design starts with the assumption that all users have basic need and limitations and that it is the designer's responsibility to understand, anticipate, and design in accordance with these needs and limitations [3].

The system approach has only recently been applied to the field of healthcare, despite successful use in other fields, including engineering, mining, nuclear power, and aviation [5]. One reason for this delayed application is the fact that most hospitals grew in direct response to social needs, without priority being given to top-down design [6]. That is, hospitals were not viewed like factories, which were often built with attention to questions of efficiently and safety. Use of system-level analyses may have also lagged behind because of the default assumption that efficiency and safety would not merit careful attention, related to the stereotype that health care professionals, and physicians in particular, are perfect[5].

After a series of egregious errors that caught the attention of healthcare professionals, the media, and the public, it became clear that something needed to be done to address the issue of patient safety [7]. In 1999, Institutes of Medicine (IOM) published a powerful report entitled, "To Err is Human," which fueled an already growing shift from viewing errors as problems of individuals to problems of systems. However, even in light of this shift, there remains a long history of a culture of blame and shame that held single individuals responsible for negative outcomes [8-10]. Active efforts will likely be required to achieve a complete shift in perspective and, in the view of some, the shift may never be complete [11-13].

Like Reason's system approach, user-centered design is also relatively new to healthcare and has been applied only to a limited degree. According to Zhang, a Health Information Technologist, "In healthcare...the culture is still to train people to adapt to poorly designed technology, rather than to design technology to fit people's characteristics" [14]. This failure of widespread adoption of user-centered design methods occurs, even despite requests for its application in a number of areas, including the development of interactive health technologies for patients [15,16]. Devito Dabbs cites the following possible reasons for the lagging adoption of user-centered methods by those in healthcare: lack of appreciation for the importance of usability testing, lack of time and resources to devote to upfront research and development, limited expertise in the principles and techniques of user-centered design, and the tendency to develop information health technologies based on developer-driven needs and priorities rather than those of the intended users [17].

Next we will explore the ways in which the system approach and user-centered design have been applied.

### A System Approach to Healthcare: Allowing for Human Nature

An eminently practical consequence of examining error with a system approach is the ability to generalize analyses to large groups of people. Whereas a person approach assumes that errors result largely from uniquely personal failures, the system approach suggests that the errors stem from identifiable patterns or tendencies, either within human beings in general or in the system's environment. The next section will outline some of the findings from this and other areas of research that illuminate relevant aspects of human behavior.

### Predictably irrational: People have systematic cognitive biases

The fields of cognitive psychology and behavioral economics, among others, have taught us that human beings are much less rational than they appear on the surface [18-20]. Yet the field of medicine has developed, in large part, as if healthcare providers, and diagnosticians in particular, were entirely rational beings. In recent years, the role of cognitive biases in medical education and training has gained some attention [21], though on the whole there still exists a relative under-appreciation of these cognitive biases among attending and trainees alike [22,23]. Some of the most common systematic information processing biases include:

• *Anchoring: *the tendency to overvalue (anchor) one piece of information when making a decision. For example, patients insufficiently adjust their subjective risk to the objective risk value communicated by healthcare providers [20,24-26].

• *Authority bias*: the tendency to overvalue the opinion of a perceived authority and undervalue one's own judgment in comparison. Studies have demonstrated that physicians sometimes tend to overvalue so-called expert opinions, in lieu of using critical analysis [25-27].

• *Availability heuristic: *making an estimate according to how easily an example can be brought to mind, while discounting more relevant information. Some studies have shown that diagnostic error can be influenced by this heuristic [25,26,28-30].

• *Base rate fallacy: *when available statistical data (base rates) are ignored in favor of one's own hypothesis [20].

• *Confirmation bias*: the tendency to search for or interpret information in a way that confirms one's preconceptions [31,32].

• *Framing*: interpreting a situation using a narrow lens [25,26,33-35].

• *Fundamental attribution error: *the tendency to over-emphasize personality variables while under-emphasizing situational variables to explain specific behaviors [36,37].

• *Hindsight bias*: the inclination to see past events as if they had been predictable without acknowledging that all of the relevant information had not been available at the time [38].

• *Illusory correlation*: an erroneous conclusion about an association that seems real but does not actually exist [39,40].

• *In-group bias*: the tendency for people to give preferential treatment to others they perceive to be members of their own groups [41,42].

• *Overconfidence effect: *excessive confidence in one's own answers to questions. An example of this is the finding that physicians, and particularly those in training, underestimate their own errors and overestimate the errors of their colleagues, even when errors in judgment are pointed out to them [22,43,44].

• *Premature closure*: the tendency to jump to a conclusion prior to having all of the relevant information, typically in order to escape the experience of doubt and uncertainty [45]

• *Representativeness heuristic*: coming to a conclusion based on how much a hypothesis resembles available data. While this is helpful in making quickly decisions in everyday life, it can result in neglect of relevant base rates [20,25,26,46].

• *Stereotyping: *when one expects a member of a group to have certain characteristics solely because of group membership, not because of individual characteristics [42].

### Shame and Blame: People hide their errors in punitive environments

Healthcare providers are reluctant to report their own errors in a system fraught with risk and blame that relies on tort law as a major regulatory force. One of the consequences of Tort law and financial markets being significant regulatory forces in the absence of non-punitive, transparent error reporting mechanisms is that clinicians and healthcare delivery systems have become reluctant to report errors, thus perpetuating high risk levels and failing to integrate corrective feedback into the system.

Clinicians are unlikely to respond to increased pressure to report within this climate, and would be more responsive to a culture that emphasizes safety and encourages learning over shaming. Learning cultures facilitate detection and sharing of errors, reflection upon and understanding of underlying causes, proactive involvement in professional life, and increased dedication to improving safety [47].

In an ideal world, clinicians would speak openly about their mistakes. Senior staff would be viewed as role models, secure about reporting and learning from their own errors. Asking for and receiving support would be encouraged and viewed as a strength rather than a weakness. Safety cultures would be strongly endorsed by top-down organizational policies and promoted by leaders as best practices.

### Physicians on a pedestal: Social power structures in healthcare

The field of medicine has been traditionally hierarchical in nature. In any hierarchical system, those with less power often find that challenging or making requests of those with more power can come at a cost. Not surprisingly, this is true in healthcare settings. As a result, when faced with the question of whether to challenge more powerful individuals, the less powerful individuals must decide which costs to incur--those resulting from a challenge or from withholding potentially corrective feedback [48]. For example, interrupting a physician by a page to report an error is a burden on physicians, who, in turn, can easily disregard the corrective feedback or not assimilate the seriousness of the error during the translation of the order into action. Correcting a charismatic physician may also lead to consequences that no one is ready to endure (e.g. pressure on the job, alienation from other members of the team, delay in promotion etc.). The result of this dynamic is that the people with less power are less likely to identify their own errors for fear of criticism or retribution, thus planting the seed of a closed loop system where little can be learned.

### Risks and benefits of teamwork: Bringing out the best and the worst

A team includes people working together to achieve shared goals. Effective teams share resources, communicate clearly, and coordinate their efforts to adapt to change [49]. Observational studies and retrospective analyses have shown that flawed teamwork rather than lack of clinical skills is a major contributor in the occurrence of errors [49,50]. Effective communication is the cornerstone around which a team is built; it should be done with trust and understanding and without fear of hierarchy. On the contrary, a leader should flatten the power distance and facilitate speaking up [51].

In healthcare, teamwork relies heavily on communication and coordination. Although healthcare providers may perceive quality of teamwork differently, they share a mental model that becomes essential to their teamwork [52]. No matter what the outcomes of new training programs, heightened public awareness and accuracy in timely diagnosis turn out to be, clinicians need to be empowered by their successful achievements as a team and supported by their organizations when they make mistakes [53]. Leadership styles that value contribution from staff will promote a climate where the information is shared in a timely manner, effectively and openly. In addition, this leadership style will increase staff well-being [54]. A healthy, happier staff is one that can shift flexibly between implicit coordination (during routine condition) and explicit coordination (during critical intervention) to ensure the highest level of patient safety [55].

### The fallacy of the health belief model: Knowing and doing are different things

Over the past 40 years advances in health psychology have demonstrated that humans often engage in behaviors that they well know to be dangerous or unhealthy. This means that health education alone, while certainly important, is likely to be insufficient to trigger behavior change. Yet, this is still the primary means of attempting to elicit behavior change in provider-patient relationships. The traditional role of the all-knowing and all-powerful physician) sets forth the implicit message, "I know what is best. I tell you (the patient) what to do and you conform." There was very little room for incorporating the experience of the patient into early models of healthcare delivery. Similarly, early health behavior change theories (e.g. Health Belief Model) assumed that if people were educated about health promoting behaviors and about consequences of high-risk behaviors, they wouldn't do them. We now recognize that human behavior is driven by a complex set of interacting variables, only one of which is knowledge about what is best for one's health [56,57]. Many of the more recent health behavior change models take more human-centered approaches that make allowances for what are now known to be common errors in logic and responses to emotional, physical, and environmental cues that distract from one's health goals. However, many elements of our current healthcare delivery systems are still founded on the premise of the less human-centered health belief model.

### Coping with uncertainty: The ultimate challenge

In his studies of human error, Reason found that uncertainty in one's environment or understanding of one's goals was a significant factor that contributed to making errors [58]. Studies of misdiagnosis have found that diagnostic uncertainty increases the likelihood that an incorrect diagnosis will be made [59]. Patients do not help in correcting the process as they also struggle in the face of uncertainty, are fearful or too trusting of authority figures [22]. In a nutshell, while the diagnostic process requires robustness and flexibility to deal with the uncertainty of not knowing or not knowing enough, physicians remain not trained nor do they tolerate uncertainty hence committing mistakes. Frequently patients will simply leave the care of a physician with whom they are dissatisfied and go elsewhere searching for certain answers. Given inadequate feedback, physicians may think that patients are not coming to the clinic because they are cured while, in truth, patients may have simply preferred not to return.

### User-Centered Design in Healthcare: A Promising Start

Early applications of user-centered design were seen primarily in medical engineering, health information management, and web design [60-63]. Safety-oriented analyses and solutions have been at the forefront of user-centered applications in healthcare, with such examples as bar-code technology [64] and checklists [65]. User-centered approaches to the study and development of provider tools have also expanded into more traditional areas of health research, including care coordination [66], data entry interface design [67,68], cognitive processes engaged during healthcare procedures [69,70], patient monitoring tools [71], and development of screening tools [72,73].

Similarly, user-centered research focused on patients has grown over the last few years, primarily in development of e-health education tools [74-76], and interactive e-health technologies [17,77,78].

Despite the promise of these emerging areas of research and practice, when considering the scope of all ongoing research, program development, and health technology innovation within the U.S. healthcare system, user-centered design is found only in a very small proportion of work being done. There are still many problems within the area of healthcare that require application of a user centered approach, including standardization of self-care tools, development of assessment and treatment tools for emotional health, chronic care tools, and preventative care systems. This paper is a call for shifting user-centered design toward the mainstream of work in these areas.

### Future applications of the system approach

While a system approach to understanding errors in healthcare has played a critical role in shifting from a Culture of Blame to a Culture of Safety, significantly more work is yet to be done in understanding the psychological and behavioral drivers of healthcare providers, patients, and other members of the system, such as family members, administrators, and those responsible for building and maintaining equipment and systems. The role of psychological defense mechanisms is not well understood and likely plays an important role in predicting areas of vulnerability for all members of the healthcare system. At the same time, a system approach to understanding how to harness and cultivate the strengths of different parts of the system is equally important to study and has been given less attention than patterns of vulnerability and weakness [79].

### Future applications of a human-centered approach

One of the most critical areas of healthcare requiring a human-centered approach is the development of standardized reporting mechanisms that, through open feedback loops, allow for the reporting of and learning from medical errors. Some of the benefits of applying a human-centered approach to the development of a safety reporting system include: an emphasis on understanding and learning over blame, attention to the existing vulnerabilities in the system, design focused on motivating people to report, an emphasis on iterative improvements, and a place for a participatory research component.

A number of Patient Safety Reporting Systems(PSRS) have emerged since 1999 with the goal of providing such mechanisms of feedback. Some are government sponsored entities, such as the PSRS developed by the VA system and NASA in 2000 and others are privately organized, most often founded by industry, professional or consumer groups.

The Patient Safety and Quality Improvement Act of 2005 is a landmark piece of legislation that provides Federal legal privilege and confidentiality protections to information reported in reference to patient safety concerns. It developed out of the recognition that, despite the importance of reporting for the purpose of learning about how and why errors occur, many healthcare providers would naturally be reluctant to report their own errors for fear of retribution. Prior to the passing of this act, studies had demonstrated that granting immunity to personnel reporting errors voluntarily would have a positive impact on the reporting incidence of errors [80].

In a non-punitive, learning culture, punishment and humiliation are replaced by an emphasis on trust and positive change. These elements are critical for maintenance and promotion of a safety culture. When healthcare professionals feel that they can report their errors without losing their jobs and reputation or fear of litigation, they will be more likely to cooperate with a root cause analysis approach to identifying and understanding errors [81].

Other areas that would benefit from application of a human-centered approach include:

• *Training in social dynamics: *Integrating knowledge of social dynamics into training of healthcare professionals and into routine team practices would be useful in creating a widespread understanding of human tendencies.

• *Accounting for patient biases: *The average patient cannot be expected to pursue positive health behaviors based only on knowledge of healthy and unhealthy behaviors. Rather, integration of education with techniques that account for known biases (e.g., motivational interviewing, behavioral economics, etc) may prove more effective in increasing the health activation levels of patients.

• *Accounting for clinician biases: *Awareness of and compensation for clinician biases can be addressed through peer consultation and application of reflective practices that incorporate knowledge of one's strengths and limitations.

• *Building resilience: *Resilience refers to the degree to which a system continuously prevents, detects, mitigates or ameliorates hazards or incidents leading to bounce back to its original ability to provide core functions following the occurrence of adverse events [82]. Taking action to reduce risk and prevent the reoccurrence of the same or incident improves system resilience [82].

## Conclusion: A sustainable solution must be human-centered at every level

An understanding of human thought processes, emotions, and behaviors needs to guide the design of healthcare delivery systems. We would be wise to apply what we know about human tendencies to build healthcare systems that optimize both patient behaviors and clinician behaviors. The more we know about how people naturally work best, the more we can leverage that to address the current problems related to patient safety [79]. All users of the healthcare system can benefit from this type of approach. Our paper is an open invitation to an overdue discussion about placing human beings in the center of our thinking about healthcare. We maintain that robust research and training efforts focused on the issues described in this paper will be critical for the evolution of a sustainable healthcare system.

## Competing interests

The authors declare that they have no competing interests.

## Authors' contributions

All authors were instrumental in the development of the initial concepts and the writing of the manuscript. All authors read and approved the final manuscript.
